# Morphology of the transverse ligament of the atlas and the alar ligaments in the silver fox (*Vulpes vulpes var*)

**DOI:** 10.1186/1746-6148-9-64

**Published:** 2013-04-04

**Authors:** Marta Kupczynska, Karolina Barszcz, Pawel Janczyk, Michal Wasowicz, Norbert Czubaj

**Affiliations:** 1 Department of Morphological Sciences, Faculty of Veterinary Medicine, Warsaw University of Life Sciences - SGGW, Nowoursynowska 159, Warsaw, 02-776, Poland; 2 Institute of Veterinary Anatomy, Faculty of Veterinary Medicine, Freie Universität Berlin, Koserstrasse 20, 14195 Berlin, Germany

**Keywords:** Atlantoaxial joint, Atlantooccipital joint, Craniocervical junction, Transverse ligament of the atlas, Alar ligament, Silver fox

## Abstract

**Background:**

Recent new anatomical and histological features of craniocervical junction in dogs and cats were described providing evidence of differences between the carnivore species. No information on these structures in foxes exists.

**Results:**

Two parts of the alar ligaments were found. A longer one aroused from dens of axis to the internal (medial) surface of the occipital condyles and was called apical part. A shorter part originated from the entire length of the lateral edge of the dens of axis and terminated on the internal wall of the vertebral foramen of atlas and thus was called the lateral part. The transverse ligament of the atlas was widened in the mid region, above the dens of axis, and thickened at enthesis. Periosteal fibrocartilage was detected in the transverse ligament of the atlas at the enthesis, and sesamoid fibrocartilage was present on periphery in the middle of the ligament.

**Conclusions:**

The craniocervical junction in foxes differs in part from other carnivores such as dogs and cats but resembles that of mesaticephalic dogs. The sesamoid and periosteal fibrocartilage supports the transverse ligament of the atlas whereas the alar ligaments have no cartilage.

## Background

The craniocervical junction (CCJ) consists of the atlantooccipital and the atlantoaxial joints and their complex arrangement of ligaments [1-3].

In the veterinary literature descriptions of the morphology of atlantooccipital and atlantoaxial joints forming the CCJ exist [1,4-8] but none of these provide details on the morphology and histology of the ligaments present in these joints in pet or fur animals. Recently a thorough description of the CCJ was reported extending our knowledge of the morphology of these structures in dogs [9]. Additionally previously unknown features in cats already have been reported [10,11].

Foxes are a group of animals having various races treated as separate subspecies. One such example is the silver fox (*Vulpes vulpes var*) acknowledged as a fixed breeding form of the American fox (*Vulpes vulpes fulva*). The first attempts of semi-free breeding of foxes were undertaken by American trappers in the 18^th^ century. However, only in the early 1890’s did the farm breeding of these animals develop. This was the result of huge interest in their skins that are renowned for their better qualities for practical uses than the skins of the wild foxes [12,13]. Selective breeding led to fixation of morphological features of these animals, mainly of their colour types. Economic reasons of breeding result in keeping the animals in cages with limited space for exercise and increased risk of injuries. Consequently intensive veterinary care is required for this species, for both farm animals and pets. The above mentioned premises are the basis of a detailed analysis of the morphology of this species, particularly in aspects that may be the direct cause of certain diseases. For example, neurological signs can occur due to dysfunction of the CCJ [14-16]. Thus, the objective of this study was to discover the morphology, topography and syntopy of the CCJ and its main stabilising ligaments, i.e. the transverse ligament of the atlas and the alar ligaments in farm bred silver fox.

## Results

The bodyweight of the skinned individuals varied between 4.60 - 5.90 kg with a mean of 5.06 ± 0.41 kg.

**Figure 1:**
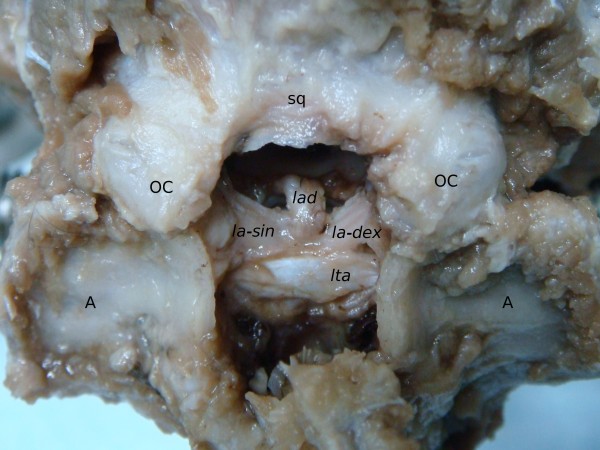
**Craniocervical junction in a silver fox. **Dorsal view of atlantooccipital and atlantoaxial joints. Dorsal vertebral arch of atlas and spinal cord were removed. Occipital squama (sq), occipital condyles (OC) and wings of atlas (A) are marked for orientation. The apical ligament of the dens (*lad*), the transverse ligament of the atlas (*lta*) and the left and right alar ligaments (*la-sin *and *la-dex*, respectively) are presented. Please notice the central widening of the *lta.*

Both the transverse ligament of the atlas and the alar ligaments are located within the spinal canal (*canalis vertebrali*s). The ligaments are clearly distinguishable and visible as stiff structures having a silvery hue (Figure 1).

The transverse ligament of the atlas stretches between the walls of the vertebral foramen (*foramen vertebrale*), on the internal surface of the atlas (C_1_). The ligament crosses the vertebral foramen and directly covers the dens of the axis (C_2_). This characteristic morphology was found in all the specimens studied. In the central region, a visible widening was observed that steadily narrowed towards its attachments on the foramen's walls (Figure 1). At their insertion points, both ends of the ligaments were slightly thickened. The length, width and thickness of the transverse ligament of the atlas were: 13.18 ± 0.46 mm; 3.50 ± 0.30 mm and 0.46 ± 0.08 mm, respectively.

**Figure 2:**
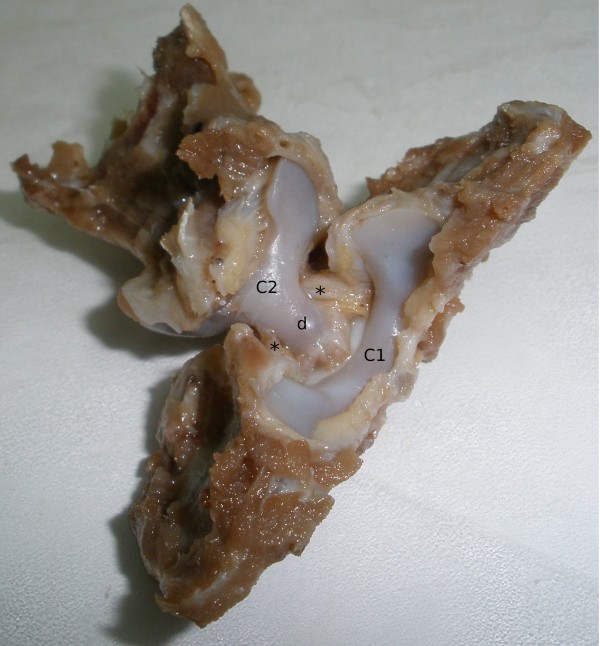
**Ventral view of atlantoaxial joint in silver fox. **Atlas (C1), axis (C2) and dens of C2 (d) are labelled for orientation. Short parts of the alar ligaments (*).

In the specimens studied features of the paired alar ligaments were determined. Each consisted of two visible parts, a long and a short one. The long part was more prominent and extended from the top of dens of C_2_ to the internal (medial) surface of the occipital condyles (*condylus occipitalis*). Considering its course, this part was determined as the apical part (*pars apicalis*). The other, shorter part, started from the entire length of the lateral edge of the dens of C_2_ and terminated on the internal wall of the vertebral foramen of C_1_ and thus was called - the lateral part (*pars lateralis*) (Figure 2). The syntopy of the apical parts of the alar ligaments may be compared to the letter V (Figure 1). Their initial area was covered by the transverse ligament (Figure 1), which, at the same time, completely covered both lateral parts. The length and width of the left and right, and the thickness of the left alar ligament were as follows: 8.59 ± 0.23 mm; 8.57 ± 0.24; 3.38 ± 0.16 mm; 3.38 ± 0.16 mm and 1.50 ± 0.02 mm, respectively.

**Figure 3:**
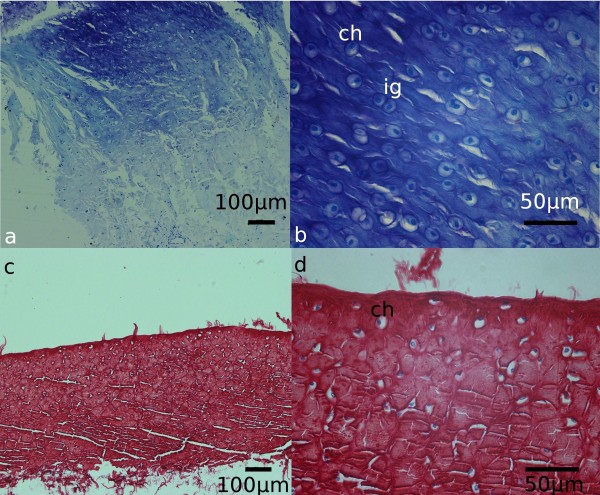
**Fibrocartilage in the transverse ligament of atlas in silver fox. **Longitudinal sections of the ligament from the enthesis are stained with toluidine blue and visualised under different magnifications **(a, b)**. Chondrocytes are found either individually (ch) or in isogenic groups (ig), between thick, parallel bundles of dense collagen, typical of periosteal fibrocartilage. Transverse sections of the central part of the transverse ligament of atlas were stained with Sirius red **(c, d)**. Under lower magnification (**c**) peripheral localisation of fibrocartilage can be observed. Under higher magnification (**d**) individual chondrocytes (ch) can be seen between collagen bundles, characteristic for sesamoid fibrocartilage.

**Figure 4:**
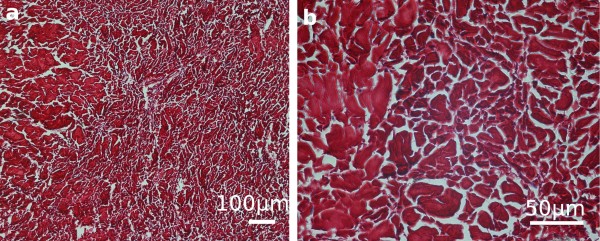
**Histological morphology of the alar ligaments in silver fox. **Transverse sections of the ligaments stained with Sirius red are presented under two magnifications **(a, b)**. Dense collagen bundles (red) can be seen and nuclei of fibrocytes (dark blue). The collagen bundles run either in parallel or cross. No chondrocytes were seen in the entire ligaments.

Histological analyses revealed differences in the structure of both ligaments mentioned above. In the transverse ligament of the atlas thick bundles of collagen fibres coursing parallel to each other were observed along its whole length. At the attachment ends and at the periphery of the central part of the ligament numerous chondrocytes lay in isogenic groups or individually, between the collagen bundles, providing evidence for the presence of fibrocartilage (*cartilago fibroidea*) (Figure 3). The alar ligaments consisted of dense collagen fibres and few elastic fibres, with fibrocytes lying among them (Figure 4). No chondrocytes could be detected there.

## Discussion

The description of the morphology of atlantooccipital and atlantoaxial joints and their ligaments in domestic animals has been presented in several publications, including anatomical monographs [4,8,17,18]. However, the cited reports treat each of the elements as a separate structure unrelated to the others. As mentioned in the introduction to this paper, these joints and their ligaments should be treated as an entity, because any disorder of one of them can result in dysfunction of the whole CCJ. A detailed morphological description of CCJ was performed in dogs [9], and some information on its features in cats have recently been reported [10,11]. The present report provides new data on CCJ morphology in other carnivore species, the fox.

The function attributed to the transverse ligament of the atlas and to the alar ligaments consists in stabilizing the dens of axis by immobilizing it in the vertebral canal and by limiting the rotation movement of the axis [19]. In this way they protect the spinal cord against pressure produced by bony elements. The present study confirmed the characteristic morphology of the transverse and alar ligaments. The features found in the fox were similar to those present in the dog [9]. However, some differences were recorded. The widening of the central region of the transverse ligament was similar to that reported for dogs of large breeds (>25 kg), medium breeds (15–25 kg) and small breeds (5–15 kg), representing the mesaticephalic dogs [10]. Thus despite the low body mass of the fox (<5 kg), considering the weight of the head in proportion to the rest of the corpus and its mesaticephalic type, the foxes can be compared to the medium sized dogs, or in general, to the mesaticephalic dogs. The widened transverse ligament above the dens can better stabilize the dens and spread the forces acting on the fibres reducing the probability of ligament rupture.

Similarly to dogs, silver foxes have a characteristic duality of the alar ligaments [9]. There is an assumption on the relationship between the development of the alar ligaments and ossification of the atlas and the axis [4]. The finding of the present study in foxes and the previous study in dogs would therefore provide further evidence for confirmation of this hypothesis. The described apical part of the alar ligaments is probably joined, in a growing potential manner, with the "proatlas center" and the lateral part depends on the "center 1" of the axis [4,9]. This hypothesis may also explain the presence in some cats of a duality of parallel streaks of the alar ligament on each side [10]. Nevertheless, without further studies applying e.g. computer tomography of the CCJ in growing animals this remains a hypothesis.

Many anatomical structures exposed to various forces are built of dense connective fibrous tissue [20]. This is characterized by few fibrocytes and intercellular substance dominated by fibres over ground substance. Considering the detailed histological structure, two types may be distinguished. The first is dense irregular fibrous connective tissue that is characterized by an arrangement of dense collagen bundles resembling plaiting, which can be accompanied by elastic fibres (in various numbers). It occurs mainly in the places exposed to stretching forces in various directions. This type of connective tissue characterised the alar ligaments investigated in the present study. The second type is dense regular fibrous connective tissue that is characterized by an ordered arrangement of parallel collagen bundles, particularly thick ones [21-23]. A similar structure may be observed in the fibrocartilage. It is characterized by rather poorly expressed ground substance (matrix) with numerous collagen fibres. They form thick parallel bundles along which there are chondrocytes lying individually or in isogenic groups, as was observed in the transverse ligaments of the atlas in the present study. Due to a large number of fibres and a low number of cells, the fibrocartilage is exceptionally resistant to forces. It is found, among others, in the intervertebral disks, pubic symphysis but most of all in the location where the ligaments and tendons are attached to bones.

The attachments of ligaments to the bone are called the "enthesis" [24]. Considering the histological structure of the enthesis, two categories are distinguishable: a fibrous and a fibrocartilaginous attachment [24,25]. The first one fixes ligaments on the shafts of long bones (*diaphyses*) and in places where epiphyseal cartilages are found. This type also characterizes the enthesis of the alar ligaments observed in foxes in the present study. When the head moves, they are only subjected to the stretching forces acting along the ligaments. Because of only few elastic fibres they remain rigid and rather inflexible thus stabilising and limiting the side rotation in the atlantoaxial joint.

The enthesis of the transverse ligament of the atlas in the foxes characterises by the presence of periosteal fibrocartilage, as it was recorded previously in dogs [9]. The fibrocartilaginous attachment, determined as periosteal fibrocartilage [26] anchors the ligaments to the base of long bones (*epiphyses*) and to the short bones [24]. The site of anchoring the ligament to the bone is a point of particular accumulation of impact forces. The presence of fibrocartilage in these places is a defence mechanism that aims at minimizing stress, pressure and stretching forces affecting especially heavily loaded ligaments. The fibrocartilage creates a brake for these forces and prevents extensive strain of the ligament. It also prevents sudden narrowing of the ligament that may bring about its rupture.

Another type of fibrocartilage, i.e. sesamoid fibrocartilage, was observed in all silver fox individuals examined in the central (widened) part of the transverse ligaments of the atlas. The sesamoid fibrocartilage, within tendons and ligaments, is observed in the sections where the direction of the ligament or tendon is changed, when these structures wrap around the bony rim or in places particularly exposed to powerful forces [9,26]. The sesamoid fibrocartilage is mainly formed in internal, concave areas of the ligament located just above the protruding bony element. Its formation is conditioned by the simultaneous action of both pressing and stretching forces [25]. This explains why this kind of fibrocartilage can be seen in the transverse ligament of the atlas just above the dens of the axis. This ligament is a subject to constant and simultaneous pressure and stretching forces. When the head moves down, the dens of C_2_ presses and raises the fragment of the ligament in this area upwards. As a result, the ligament is strained and, at the same time, the second force exerts pressure perpendicularly to the first one and along the ligament. It may be presumed that, as in dogs, the fibrocartilage protects and enables slipping of the transverse ligament on the dens of C_2_[9].

## Conclusion

The craniocervical junction in silver foxes consists of most features present in other carnivores and resembles the CCJ of mesaticephalic dogs. The transverse ligament of the atlas consists of sesamoid and periosteal fibrocartilage, whereas no cartilage supports the alar ligaments.

## Methods

The study was performed on 15 corpses of adult (3–5 years old) silver fox males. Foxes were provided by a commercial fox farm (Polish veterinary accession nr. 14059001) where they were killed for fur by qualified personnel by electrical stunning according to the European law [27] and under the control of the Veterinary Inspection legislation [28]. As the animals were not killed for the purposes of the study, no additional ethical authority permission was required. Using such cadavers reduces the number of animals used for morphological research.

The skinned cadavers were chilled and transported to the anatomical theatre where they were fixed in 10% formaldehyde. The body weight of the specimens was recorded after skinning. The atlantooccipital and atlantoaxial joints were dissected thoroughly, with particular attention being paid to the transverse ligament of the atlas *(ligamentum transversum atlantis*) and to the alar ligaments (*ligamenta alaria*). The morphological analysis was carried out with the use of an ECLERIS HALOLUX 150 operating microscope. The studied ligaments were exposed by excising the dorsal arch of the atlas and removing the spinal cord and the covering membrane. At this stage of the dissection, the length and width of the transverse ligament were measured. Next, the transverse ligament of the atlas was cut off at its attachments sites and its thickness was measured at the central part. After removing the transverse ligament of the atlas, the dens of axis and the terminal attachments of the alar ligaments were exposed. At this stage the length of the left alar ligament and of the right alar ligament and their width were measured. Later, the left alar ligament was dissected and its thickness was measured in its central part. The measurements were performed with a Digital caliper with 0.01 mm resolution. Photographic documentation was also performed for further stages of dissection.

The dissected ligaments from all 15 specimens were taken for histopathological analysis. The transverse ligament was cut in the median plane and its parts were analyzed both in transverse and longitudinal sections. The left alar ligament was only sectioned transversely. Following standard paraffin embedding, serial sections, 7 μm thick, were stained with standard hematoxylin and eosin, with Sirius red to visualise collagen fibers, with orcein for elastin fibers and with toluidine blue for cartilaginous tissue [29].

All morphological terms used in the study are conforming to the current anatomical nomenclature [30].

## Competing interests

Authors declare no competing interests.

## Authors’ contributions

KB, MW, NC carried out the dissection of the cadavers and measurements, and performed the histological analyses. MK conceived of the study, and participated in its design and coordination and helped to draft the manuscript. PJ participated in the design of the study, analyses of the specimens and histological slides, and helped to draft the manuscript. All authors read and approved the final manuscript.
